# Supplementary material for: A comparative study of thermal radiation model in Chinese solar greenhouse
Source: PLoS One. 2024 Sep 13;19(9):e0309225. doi: 10.1371/journal.pone.0309225 (PMC11398644; doi:10.1371/journal.pone.0309225)
Supplement: Attachment — Submitted filename: PONE-D-23-28972_Rev.pdf [file pone.0309225.s002.pdf]

Review of the manuscript titled: A Comparative Study of Thermal Radiation Model in Chinese Solar Greenhouse

The radiation models including P1, DO and S2S were applied to perform the thermal analysis of the Chinese solar greenhouse. The accuracy of radiation models were investigated to predict the temperature of the rear wall, soil and air inside the heliostat and the results were compared with experiments. This work provides information for selection of the proper radiation model of CSG thermal analysis. The precise simulated temperature distribution of CSG can be used for designing the suitable thermal environment for crop growth. However, the optical boundary conditions were not defined and the models differences were not properly discussed. The novelty of this work is not clear too. The results are useful for practical purposes and the draft can be considered as technical note.

The following modifications should be applied.

1. Which radiation model was used in Ref [10] CFD model?
2. What is the meaning of “radiative effective” mentioned from Ref [12] results?
3. The turbulence models should be typed like formulas and “by compared with” needs revision in the Ref [14] results.
4. The sentence “Even if the using of CFD has been widely expanded to analysis this problem in depth.” should be modified.
5. Generally, section 2, first paragraph “Experimental method and theoretical considerations” needs a revision.
6. The mesh convergence study should be added.
7. The sentence “The outdoor temperature is timely by TRM-ZSF GPRS wireless remote control system of Jinzhou Sunshine Technology Co., Ltd. Monitors”, requires correction.

8. According to Fig. 4, what is the logic of temperature probes arrangement and why 8 probes were selected? What is the reason for putting the ambient temperature probe at that specific location? Did it sense the outside wind effect?
9. The governing equations should be mentioned and the relation between source term of energy equation with radiation models should be described.
10. The sentence “The large scale turbulence and the widest application range in industrial flow and heat transfer simulation are reasonably”, needs revision.
11. The authors need to explain how the ideal gas can be considered as incompressible and with this assumption, the buoyancy effect can be simulated.
12. What does this sentence mean?” The DTRM radiation model is applicable to all optical thickness models, but it is rarely used because it cannot be solved”.
13. The explanations about S2S radiation model should be more carefully rewritten.
14. The number of discretization in each direction for DO model should be mentioned and their independency should be studied.
15. The optical boundary conditions for modeling radiation, convention and conduction are required which are the main parts that should be added.
16. What is the reason of differences between radiation models results and the experiments?
17. “is lowed” should be corrected with “is reduced”.
18. According to Fig. 11, the peak of t3 is higher than t4 which is not observed in the temperature contours of figures 13,14 and 15. Please describe the reason.
19. The manuscript should be spell checked and grammar checked again.
