# Supplementary material for: A comparative study of thermal radiation model in Chinese solar greenhouse
Source: PLoS One. 2024 Sep 13;19(9):e0309225. doi: 10.1371/journal.pone.0309225 (PMC11398644; doi:10.1371/journal.pone.0309225)
Supplement: Attachment — Submitted filename: PONE-D-23-28972_R2.pdf [file pone.0309225.s004.pdf]

Comments from the Reviewers (PONE-D-23-28972_R1_reviewer)

Most of the grammar and spelling errors have been corrected; however, the authors have not addressed the following points.

- In question 5, the experimental set up were explained and it was mentioned that different materials were used in the solar greenhouse walls which their thermal and radiative boundary conditions were not given in Table 2. Although some assumptions can be made and average boundary conditions can be used; however, none was given in Table 2.

- In question 14 regarding the discretization in each direction for DO model, it was mentioned that the value for theta division was 8 and phi was 2 which are not enough at all. Then, the results of DO model are not reliable based on the author's response.
