# Supplementary material for: A comparative study of thermal radiation model in Chinese solar greenhouse
Source: PLoS One. 2024 Sep 13;19(9):e0309225. doi: 10.1371/journal.pone.0309225 (PMC11398644; doi:10.1371/journal.pone.0309225)
Supplement: Attachment — Submitted filename: Response to reviewers 2.0.docx [file pone.0309225.s005.docx]

Response to reviewer #1

We sincerely thank the editor and all reviewers for their valuable feedback that we have used to improve the quality of our manuscript. According to your nice suggestions, we have made extensive corrections to our previous manuscript, the detailed corrections are listed below.

Most of the grammar and spelling errors have been corrected; however, the authors have not addressed the following points.

- In question 5, the experimental set up were explained and it was mentioned that different materials were used in the solar greenhouse walls which their thermal and radiative boundary conditions were not given in Table 2. Although some assumptions can be made and average boundary conditions can be used; however, none was given in Table 2.

The Table 2 was modified as reviewer’s advice, which is shown as follow:

**Table 2.**Optical parameters of materials in greenhouses

| materials/position | Boundary type | Absorption coefficient(m^-1^) | emissivity | absorptivity | transmissivity |
| --- | --- | --- | --- | --- | --- |
| air | transparent | 0.50 | - | - | - |
| Greenhouse film | semi-transparent | - | 0.85 | 0.95 | 0.95 |
| Soil surface | opaque | - | 0.95 | 0.6 | - |

their thermal and radiative boundary conditions for greenhouse simulation are set in combination with actual measurement conditions. The external temperature values of the greenhouse at different times are used as the environmental convection boundaries for the enclosure structure and outdoor soil. The temperature values are shown in Fig. 6. Using temperature values at different times as the environmental convection boundary of the greenhouse film, the temperature values are shown in Fig. 7. In order to solve the heat transfer process of solar greenhouse under 24h sunlight, the solution step is set as 1800s, and the total solution time is 86400s.

- In question 14 regarding the discretization in each direction for DO model, it was mentioned that the value for theta division was 8 and phi was 2 which are not enough at all. Then, the results of DO model are not reliable based on the author’s response.

According to the reviewer’s require, the value of DO model for theta division was modified as 8 and phi was modified as 5. Therefore, the results of DO model are reliable, which is modified in manuscript.
